# Plant sterols, marine-derived omega-3 fatty acids and other functional ingredients: a new frontier for treating hyperlipidemia

**DOI:** 10.1186/1743-7075-7-76

**Published:** 2010-09-28

**Authors:** Christopher PF Marinangeli, Peter JH Jones

**Affiliations:** 1 Richardson Centre for Functional Foods and Nutraceuticals. University of Manitoba. 196 Innovation Drive, Winnipeg MB, R3T 2N2, Canada

## Abstract

As hyperlipidemia, including hypercholesterolemia (HC) and hypertriglyceridemia (HTN), continue to challenge North America's healthcare systems, patients continue to seek efficacious and safe natural therapies that complement pharmaceutical interventions. However, despite the ever-growing body of research supporting the use of functional foods and nutraceuticals (FFN) for the prevention and treatment of hyperlipidemia, reception amongst the medical community regarding the implementation of FFN into clinical guidelines continues to lag. Research demonstrates that specific FFN target and modulate molecular processes that perpetuate hyperlipidemia. In addition, studies consistently demonstrate that combining certain FFN such as marine-derived omega-3 fatty acids or plant sterols/stanols with statins enhances triglyceride and cholesterol-lowering efficacy, respectively. Thus, the purpose of this commentary is to contend that efficacious FFN not only reduce HC and HTG but also boost the lipid-lowering effects of pharmaceutical hypolipidemic medications. Finally, this editorial aims to challenge current medical guidelines to emphasize efficacious FFN during all stages of treatment of hyperlipidemias as adjuncts to pharmacotherapy.

## Introduction

Hyperlipidemia, including hypercholesterolemia (HC) and hypertriglyceridemia (HTG), remains a formative burden on the health care systems of North America. Given that a third of American adults meet the criteria for metabolic syndrome [1], recent studies suggest extraordinarily high prevalence rates of HC and HTG amongst metabolic syndrome patients at 69 and 65%, respectively [2]. In addition, hyperlipidemias are risk factors for vascular disease such as atherosclerosis [3,4]. Lifestyle interventions including diet, exercise and weight loss are primary strategies during the initial stages of treatment of HC and HTG [3,5]. However, if lifestyle strategies are ineffective and/or patients begin to exhibit multiple risk factors for chronic disease, healthcare practitioners turn to lipid-lowering pharmaceuticals [5,6]. That being said, functional foods and nutraceuticals (FFN) represent a growing genre of therapies which have demonstrated efficacy for hyperlipidemia when administered alone or in combination with pharmaceutical treatments. Nonetheless, the question remains, where should FFN be positioned in current guidelines as treatments for HC and HTG?

Over the last decade, considerable focus amongst the research community has been directed toward enhancing our understanding of FFN as therapies for lifestyle-related diseases. As the severity of symptoms worsen and patients advance through various stages of treatment, prescription medications become the primary therapeutic strategy, discounting FFN as disease-thwarting agents. Hypercholesterolemia, characterized by escalating LDL-C levels, is a good example. The National Cholesterol Education Program (NCEP) describes the use of plant sterols/stanols (PS) as a therapeutic option to physicians for reducing circulating LDL-C levels. Despite the plethora of peer-reviewed articles demonstrating consistent reductions in LDL-C with PS use, the NCEP does not include PS fortified foods as a constituent of the Therapeutic Lifestyle Diet, nor are PS emphasized during the latter stages of therapy when pharmaceuticals have been introduced to treatment regimens [5]. Similarly, marine derived omega-3 fatty acids (MOM-3), fibers, nuts, and soy proteins have all been shown to significantly reduce clinical endpoints associated with HTG and HC and as with PS, have not been adopted into treatment regimens either. In addition, research demonstrates that therapeutic FFN, such as PS and MOM-3, can complement pharmaceutical treatments, producing better clinical outcomes than pharmaco-mono-therapy [7].

**Figure 1:**
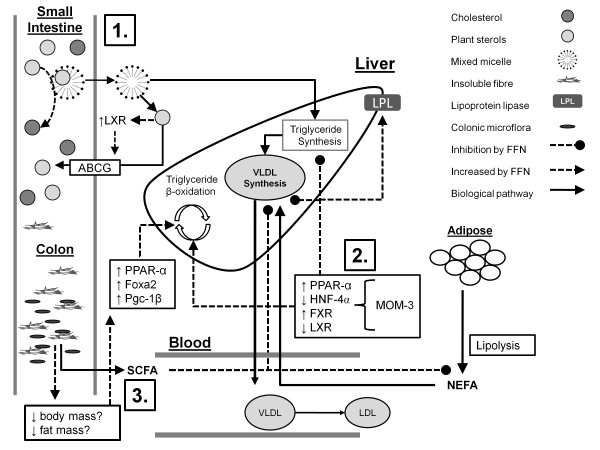
**Mechanisms by which plant sterols, marine-derived omega-3 fatty acids, and insoluble fibres decrease dyslipidemia**. **1**. Plant sterols (PS) displace dietary cholesterol from mixed micelles. Absorbed PS activate liver X receptor (LXR), increasing the expression of ATP-binding cassette G transporters (ABCG) which pump absorbed PS back into the intestinal lumen. **2**. Marine-derived omega-3 fatty acids (MOM-3) increase the expression of peroxisome proliferator-α (PPAR-α) and farnesol X receptor (FXR) while reducing the expression of hepatocyte nuclear-4α (HNF-4α) and LXR. Altogether, modulation of transcription factors by MOM-3 increases triglyceride β-oxidation and expression of lipoprotein lipase (LPL) alongside a decrease in triglyceride synthesis. **3**. Short-chain fatty acids (SCFA) produced from colonic fermentation of insoluble fibres decrease adipose lipolyisis, reducing circulating levels of non-esterified fatty acids (NEFA). Insoluble fibers have also been shown to inhibit excessive body fat accumulation which is hypothesized to propagate the expression of hepatic forkhead transcription factor (Foxa2), peroxisome proliferator-activated receptor (PPAR)-γ coactivator β (Pgc-1β), and PPAR-α, increasing hepatic triglyceride β-oxidation.

**Figure 2:**
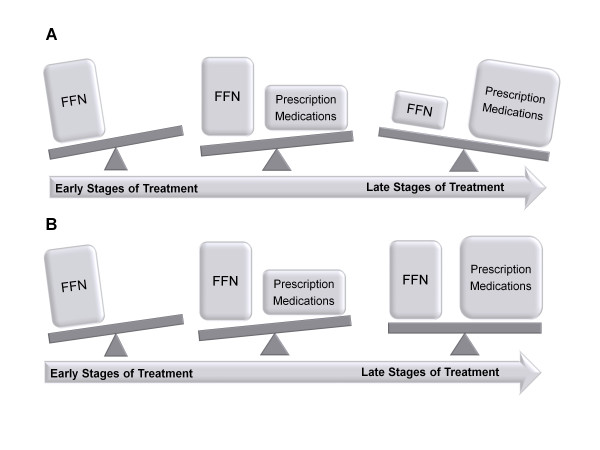
**Functional foods and nutraceuticals (FFN) as adjuncts to pharmacotherapy for the treatment as hyperlipidemia**. **Part A **is a visual depiction of how current clinical guidelines de-emphasize the use of (FFN) as treatments for hyperlipidemia progress. **Part B **incorporates the notion that FFN should be emphasized during all stages of treatment for hyperlipidemia.

Functional foods and nutraceuticals are not merely lifestyle interventions. Similar to pharmaceutical agents, FFN contain bioactive substances that, when administered at therapeutic doses, target and modulate biological processes that foster the development of disease (Figure 1). Thus, the gap that currently exists between FFN research and the medical community needs to be closed such that FFN can be implemented into clinical guidelines so that treatments for hyperlipidemia can be optimized throughout all stages of therapy (Figure 2).

### Clinical Efficacy of Functional Foods and Nutraceuticals for Treating Hyperlipidemia

Functional foods house bioactive compounds that elicit biological effects beyond that of providing nutrition. Typically functional foods can be consumed as whole foods or their bioactives can be extracted and added to other foods or concentrated into tablets, capsules or elixirs as nutraceuticals. The disease modulating effects of FFN have been known for decades. Over 50 years of research demonstrate that 1.5-2.5 g/d PS can reduce circulating LDL-C levels up to 15% [8-10]. The positive correlation between PS-derived LDL-C lowering efficacy and baseline LDL-C levels [11] indicate that the ability for PS to lower LDL-C actually improves as LDL-C levels escalate. Thus, individuals demonstrating the greatest risk of cardiovascular disease secondary to high LDL-C levels benefit the most from PS therapy. In addition, prospective analysis of men at high risk for cardiovascular disease revealed that a high PS-to-cholesterol ratio significantly reduced the relative hazard risk for mortality at 0.51 (95% CI, 0.30-0.87) compared to those with a low PS-to-cholesterol ratio [12].

Similar to PS, the triglyceride-lowering effects of MOM-3 have been thoroughly researched [13,14]. Studies have consistently demonstrated that 2-4 g/d supplemental MOM-3 reduce circulating triglycerides up to 34% in hypertriglyceridemic patients [15]. The benefits of MOM-3 supplementation have also translated into reductions in hard endpoints. Results from the GISSI-prevenzione study showed that four months of supplemental MOM-3 at 1 g/d in myocardial infarct patients demonstrated a 50% reduction in the risk of mortality [16]. After 3.5 years, the overall risk of death and cardiovascular-related death was decreased 20% and 30%, respectively [17]. Finally, a large clinical trial (n = 18 645) with patients randomized to receive 1.8 g/d MOM-3 noted a 20% reduction in major coronary events over 5 years [18]. Research demonstrates that MOM-3 are not only potent triglyceride-lowering agents, but also useful in reducing the risk of mortality.

Based on sound clinical research, effective dietary regimens incorporating soy protein, dietary fiber, PS and nuts have also been explored. When combined, constituents of this "Portfolio Diet" were shown to reduce LDL-C levels to the same extent as statin therapy at 29% and 31%, respectively [19]. In addition to a 30% reduction in LDL-C levels, subjects consuming the Portfolio Diet demonstrated a decrease in small dense LDL particles (sdLDL) [20], a risk factor for atherosclerosis given their heightened susceptibility to oxidation and ability to penetrate arteriole walls [21-23]. Moreover, when the effects of the Portfolio Diet and statin therapy on sdLDL were compared, the main site for reductions in circulating LDL-C levels for both strategies were attributed to a decrease in cholesterol found in the sdLDL subfraction at -0.69 mmol/L and -0.99 mmol/L, respectively [24]. Overall, MOM-3, PS and the Portfolio Diet highlight the medicinal potential of FFN as therapies for combating HC, HTG and subsequent deterioration of vascular function.

### Functional Foods and Nutraceuticals Target Biological Processes that Modulate Clinical Endpoints

FFN differ from most traditional diet-based lifestyle interventions because most dietary recommendations involve the limiting of factors that promote the development of hyperlipidemia such as saturated fat [5]. FFN therapies on the other hand, assume a similar approach to pharmaceutical therapies and involve the introduction of bioactive compounds that target and positively modulate biological processes (Figure 1). For example, fibrates, a category of common lipid-lowering medications, act as ligands for transcription factors, enhancing peroxisome proliferator-activated receptor alpha (PPAR-α) activity, while reducting hepatocyte nuclear-4α (HNF-4α) activity. The result is an increase in lipoprotein lipase expression and fatty acid oxidation, alongside a reduction in VLDL synthesis [25,26]. Supplemental MOM-3 impose similar effects on triglyceride synthesis and oxidation via modulation of PPAR-α and HNF-4α expression. In addition, MOM-3 also increase and decrease farnesol X receptor (FXR) and liver X receptor (LXR) expression, respectively, causing a down regulation in the expression of sterol-regulatory binding protein 1c (SREBP 1c) [27]. SREBP 1c up-regulates the expression of genes involved in TG synthesis [28]. Overall, studies demonstrate that MOM-3 are similar to common prescription medications in that they modulate the biological processes that regulate TG metabolism.

Plant sterols' cholesterol-lowering mechanism of action is inhibition of dietary cholesterol absorption in the small intestine by displacing exogenous cholesterol from mixed micelles. Studies demonstrate that PS act as agonists for LXR and subsequently increase the expression of ATP-binding cassette G (ABCG) transporters within the epithelial membrane [29]. ABCG transporters prevent excessive absorption of PS by pumping intracellular PS from epithelial cells back into the intestinal lumen [30]. A relatively small proportion of individuals possess a genetic condition called sitosterolemia whereby high levels of PS are absorbed and are atherogenic. Thus, an evolutionary presence of PS within the diet is suggested by the fact that PS facilitate their own removal from the body, where high levels of PS are not permitted to accumulate in the blood.

In addition to impaired glucose metabolism, insulin resistance propagates dyslipidemia via increased de novo lipogenesis and high levels of circulating VLDL and LDL particles [31]. Typically, insulin resistance is linked to obesity, fostering adipose lipolysis and increased levels of circulating non-esterified fatty acids (NEFA). NEFA induce insulin resistance by inhibiting intracellular signaling processes downstream of insulin/insulin-receptor interactions [32]. Dietary insoluble fibres have been linked to reductions in insulin resistance [33,34]. Although mechanisms are not completely understood, it is hypothesized that colonic fermentation of insoluble fibre produces short-chain fatty acids (SCFA) which subsequently reduce lipolysis and circulating NEFA [35]. Recently, Roberston et al. [36] demonstrated that 30 g/d supplemental resistant starch, a type of insoluble fibre, increased insulin sensitivity alongside SCFA uptake by muscle and adipose, circulating levels of ghrelin and expression of adipose lipoprotein lipase. A recent comparison of the metabolic effects of insoluble and soluble fibre in mice revealed that, in addition to lower insulin resistance, mice fed insoluble fibre demonstrated lower body weight and fat mass despite no differences in energy intake [37]. Expression of G-protein coupled receptor 40 (GPR40) in adipose tissue was also lower in mice fed insoluble fibre. Decreased expression of GPR40 has been linked to reduced obesity-induced insulin secretion by pancreatic β-cells [38]. Finally, the present study noted increased expression of transcription factors that are associated with triglyceride beta oxidation including hepatic forkhead transcription factor (Foxa2), peroxisome proliferator-activated receptor (PPAR)-γ coactivator β (Pgc-1β), and PPAR-α [37]. The authors attribute enhanced expression of transcription factors to the effect of insoluble fibre on inhibiting excessive weight gain and body fat accumulation. Increased lipid oxidation was reflected in liver homogenates demonstrating lowering triglyceride levels in mice fed insoluble fibre compared to soluble fibre [37]. Research demonstrates that insoluble fibres modulate hepatic and adipose tissue metabolism, reducing insulin resistance as well as processes that lead to diabetic dyslipidemia. FFN need to be viewed as treatments that can profoundly benefit hyperlipidemic-related disease outcomes.

### Functional Foods and Nutraceuticals as Adjuncts to Pharmaceutical Therapies

The purpose of this commentary is not to discourage the use of pharmaceuticals. Such interventions are an invaluable part of global healthcare systems. The present aim is to emphasize that not only do specific FFN target biological processes that propagate hyperlipidemia, but that certain FFN can serve as beneficial adjunctive treatments which enhance pharmacotherapy. Combining MOM-3 and/or PS with statin therapy has been shown to reduce triglyceride and LDL-C levels by an additional 15% and 17%, respectively [39,40]. Moreover, PS/statin treatments can lower LDL-C levels equivalent to a double dose of statins (-39%) [41]. Comparing 40 mg/d simvastatin to therapeutic lifestyle interventions that combined MOM-3 and Chinese red yeast rice noted similar reductions in LDL-C levels between groups at 39 and 42%, respectively [42]. However, subjects consuming MOM-3 and red yeast rice also noted a 29% reduction in triglyceride levels, an observation not demonstrated with statins alone. We acknowledge that Chinese red yeast rice is a natural source of lovastatin [43]. However, the amount of Chinese red yeast rice-derived lovastatin provided in the present study was only 10-15 mg/d, far less than a therapeutic dose of prescription statin [42]. This suggests that PS alongside a low-dose of statin may provide the same LDL-C-lowering efficacy as typical statin treatments minus the side-effects. Observations that FFN/prescription therapies produce additive or synergistic effects are encouraging. However, a need continues to exist for pertinent developments in FFN research to be openly communicated to healthcare practitioners so that they may be implemented into current clinical guidelines and utilized by physicians to treat hyperlipidemia.

### Future Directions for Incorporating Functional Foods and Nutraceuticals into Hyperlipidemic Clinical Guidelines

Clearly, not every identified bioactive that elicits a beneficial effect on blood lipid levels will be a candidate for inclusion into medicinal treatment guidelines. Dosage, reproducible efficacy, and especially safety, must be thoroughly examined prior to their approval and implementation. However, FFN such as PS and MOM-3, which together amount to a surplus of safety, efficacy and mechanistic studies, need to be examined as suitable candidates as late-stage adjuncts to prescription medications as treatments for dyslipidemia. Moreover, similar to medications, patients must be individually assessed as to whether they would benefit from FFN/prescription regimens. For example, patients diagnosed with sitosterolemia, as described above, would obviously not be suitable for PS therapy. Other strategies such as the Portfolio Diet, which includes PS, could likely be implemented into patients' hypolipidemic treatment regimens with minimal concern considering many components of the Portfolio Diet such as soy proteins, fibre and nuts are common dietary constituents. Increasing the presence of FFN within clinical guidelines requires systematic evaluation of candidate FFN and these processes are beyond the scope of this commentary. Nonetheless, the advent of combination FFN/prescription therapies will require that physicians undergo additional nutritional training and likely enhance dietitians' role in executing patient treatment regimens, especially when whole foods are utilized as vehicles for administering FFN.

## Conclusions

Despite clinical studies showing that therapeutic dosages of FFN effectively target and modulate biological processes that foster the development of hyperlipidemia, FFN continue to be overshadowed by prescription medications as patients progress through consecutive stages of treatment. Research demonstrates that specific FFN are efficacious adjuncts to pharmacotherapy for the treatment of hyperlipidemia. Hence, it is imperative that developments in FFN research are incorporated into current clinical guidelines that are used for treating HC and HTG. In the wake of current prevalence rates of hyperlipidemia amongst people with metabolic syndrome FFN can serve as efficacious adjuncts to pharmo-therapy during all stages of treatment.

## Abbreviations

ABCG: ATP-binding cassette G transporter; FFN: functional foods and nutraceuticals; Foxa2: hepatic forkhead transcription factor; FXR: farnesol X receptor; HC: hypercholesterolemia; HNR-4α: hepatic nuclear receptor-4 alpha; HTG: hypertriglyceridemia; LDL-C: low density lipoprotein-cholesterol; LPL: lipoprotein lipase; LXR: liver X receptor; MOM-: marine-derived omega-3 fatty acids; Pgc-1β: peroxisome proliferator-activated receptor (PPAR)-γ coactivator β; PPAR: peroxisome proliferator receptor; PPAR-α: peroxisome proliferator receptor alpha; PS: plant sterols and/or plant stanols; VLDL: very low density lipoprotein.

## Competing interests

The authors declare that they have no competing interests.

## Authors' contributions

CPFM and PJHJ equally contributed to conceptualizing and writing the present commentary. Both authors have read and approved the final manuscript.

## Authors Information

Christopher P.F. Marinangeli is a PhD candidate at the University of Manitoba's Richardson Centre for Functional Foods and Nutraceuticals. The focus of Mr. Marinangeli's research is evaluating the efficacy of functional food ingredients on indices of cardiovascular disease and diabetes.

Dr. Peter J.H Jones, holds a Canada Research Chair in Functional Foods and Nutrition at the University of Manitoba. Dr Jones serves as Director of the Richardson Centre for Functional Foods and Nutraceuticals. Dr. Jones is a professor in the Departments of Food Science and Human Nutritional Sciences. Dr Jones' research interests cover cholesterol, fat and energy metabolism. Dr. Jones has published over 250 peer-reviewed research articles and reviews in international journals, as well as chapters in leading nutrition textbooks.
